# Habitual prospective memory in preschool children

**DOI:** 10.1371/journal.pone.0293599

**Published:** 2023-10-31

**Authors:** Andrew J. Kelly, Abigail A. Camden, Melany C. Williams, Michael J. Beran, Bonnie M. Perdue

**Affiliations:** 1 Department of Psychology, Georgia State University, Atlanta, GA, United States of America; 2 Department of Psychology, Agnes Scott College, Decatur, GA, United States of America; Carnegie Mellon University, UNITED STATES

## Abstract

Habitual prospective memory (PM) refers to situations in which individuals have to remember to perform a future task on a regular and frequent basis. Habitual PM tasks are ubiquitous and the ability to successfully complete these tasks (e.g., remembering to bring your lunch to school every day) is necessary for children as they begin to establish their own independence. The current investigation is the first to explore preschool children’s ability to complete this kind of task. At the end of a regular testing session during which children engaged in a variety of unrelated cognitive tasks, participants were instructed to ask for a stamp on their card, which was sitting in a box on the table. Over the course of the first experiment, participants did this 13 times, spanning a time period of several months. The results demonstrated that children initially needed prompting from the experimenter to remember, but with experience, participants were able to retrieve this intention without assistance. Experiment 2 demonstrated that removing the box from participants’ line of sight after numerous opportunities to perform the task did not negatively impact performance, although it did make a difference at the outset of this requirement to remember to ask for stamps. Together, these results indicate that with somewhat consistent and repeated practice, preschool children can fairly quickly demonstrate the ability to successfully perform future intentions that are likely to be repeated on numerous occasions.

## Introduction

Prospective memory (PM) involves forming an intention that cannot be acted on immediately but must be remembered at a specific future time or place [1]. The ability to successfully plan and complete future intentions may be a particularly important skill for young children to develop as they gain independence. For instance, teachers will expect that school-aged children remember to complete their homework every night and return it. That seemingly simple behavior, which will become routine for most students, involves many complex steps. The goal of the current study was to carefully explore the manner in which this kind of prospective remembering might develop in children, including factors which may promote or impair remembering.

### Theoretical accounts of prospective memory and its development

The most common type of experimental PM tasks assess event-based PM. In these kinds of tasks, participants engage in an ongoing task and receive specific instructions that when they encounter a particular event, referred to as the PM target, they should make a special response (i.e., PM response) instead of responding to the ongoing task [2]. For example, Kvavilashvili and colleagues [3] devised an ongoing task in which children identified a variety of objects represented in line drawings. The children were also given PM instructions to place animal drawings in a particular box to help their “friend” Morris the Mole, who was scared of animals. This was the PM intention that had to be encoded and then retrieved when the correct PM target presented itself.

Successful PM retrieval is thought to depend on controlled monitoring, spontaneous retrieval, or both. Controlled monitoring is a resource-intensive strategy that involves first establishing an intention and then initiating a “retrieval mode” in which an individual expends some amount of cognitive resources to monitor the environment for a target or target-related information [4]. Evidence of controlled monitoring can be observed in reaction time (RT) costs in the ongoing task and in the increase in PM failures associated with increased demands of an ongoing task [5]. Spontaneous retrieval has been described as an automatic or reflexive/associative process that is resource-inexpensive relative to controlled monitoring [4]. Observations of successful PM retrieval in the absence of RT costs [6] support the idea that PM retrieval can, under certain conditions, occur spontaneously.

In terms of development, PM ability improves across childhood and into adolescence, peaks in early adulthood and declines later in life [7–13]. Because this developmental trajectory closely mirrors that of executive functions (EF), Mahy and colleagues posited that working memory, inhibition, shifting, and monitoring are essential factors that contribute to the development of PM [11]. Briefly, the executive framework of PM suggests that PM performance in childhood will depend directly on the child’s ability to recall the intention (i.e., retrospective memory) and the skills necessary to implement the intention while managing distraction from the ongoing task (i.e., executive functions). This model predicts that individuals with poor EF will show poor PM performance, and that PM performance should improve as EF develops. While development in other domains are also relevant to PM performance (see Cottini [14] for a review), the majority of research with children has focused on how cognitive resource demands and availability affect performance.

Given that PM performance in children appears highly dependent on available cognitive resources, it would be expected that variables that manipulate the need to use these resources would impact PM performance. For example, Cottini recently reviewed research on the effectiveness of visual reminders for children and found that these reminders consistently improved PM performance [14]. Her analysis suggests that by having a clearly visible representation of the intention, visual reminders free up cognitive resources, thereby reducing the risk of failure due to resource depletion when attention is allocated elsewhere.

Research on the salience of the PM target relies on a similar logic. Salient cues are recognized more easily and therefore, those EF resources needed for cue detection can be used in other ways. In the context of prospective remembering, salience and reminders function similarly in that more salient cues are recognized more easily (as are reminders), and thus are more likely to produce higher prospective remembering. In preschool children, there have been several investigations that point to highly salient targets being associated with better performance [9, 15]. Kretschmer-Trendowicz and Altgassen had individuals from a lifespan sample perform a categorization task [10]. In the low salience PM condition, the target items appeared during the task in the same manner as all other ongoing task stimuli. In the high-salience PM condition, the target items appeared during the task with a red frame around them. This red frame only happened when the targets appeared, making this event stand out. The finding most relevant to the current investigation was that children in the high-salience PM condition responded correctly to more targets than those in the low-salience condition. Thus, salience and visual reminders for PM for preschool children support prospective remembering by allowing children to use their cognitive resources elsewhere. When reminders are not available or the PM target is not salient, more resources are needed, which may ultimately hinder successful PM performance, as it does for adults.

### Practice and habitual PM

While the role of EF has been established in the development of PM, the role of experience and practice has not been examined as carefully. The primary paradigm through which the effects of practice have been assessed is known as the habitual PM paradigm. Einstein and colleagues were the first to establish this paradigm [16]. In their study, older and younger adults completed 3 minutes of an ongoing task with the instruction to press the F1 key once (and only once) during that 3-minute interval. The three-minute interval was repeated for 11 consecutive blocks. As predicted, young adults outperformed older adults, and performance remained stable throughout the task. More recently, research has assessed the mechanisms that transition a PM task from more event-based to habitual. Vogel and colleagues had undergraduates complete over 700 trials (across 7 blocks) of an ongoing shape-matching task [17]. Some participants were given PM instructions to respond to particular shapes during this task, which should be a resource demanding requirement. The PM target occurred 63 times. PM accuracy increased with practice (see Meier et al. [18] for a similar result). Importantly, RT costs for the ongoing task decreased across blocks. Together, this indicates that elements of the PM task such as cue detection or ongoing task performance become more automatic with practice. This is a critical result as current theories of PM and PM development would predict that a resource-demanding PM task would remain so regardless of how many times a PM target is encountered. Therefore, practice and experience are clearly an important yet unexplored aspect of PM.

To the best of our knowledge, no studies have examined habitual PM in children and very few studies have tested children on more than one occasion. Kelly and colleagues had preschool children sort pictures of varying sizes into large-size and small-size piles with a PM instruction to sort any pictures of animals into “zoos,” which were cardboard boxes on the table in front of them [19]. Participants completed three sessions of this task, one session per week with new targets each time. Children showed improvement across sessions. In the first session, there was a significant positive correlation between age and PM performance such that older children performed better on the task. However, after repeated testing, that significant correlation between age and PM performance was eliminated, indicating differences in cognitive abilities (e.g., EFs) due to age were no longer relevant after repeated practice.

### Suspended intentions

Another avenue to explore the automaticity of a PM intention is through the use of canceled or suspended intentions. In these experiments, participants are given standard instructions to make a particular PM response to particular target items during the ongoing task. After some time working on the task with active PM intentions, participants are then told to continue working on the ongoing task, but that they do not need to perform the PM task because it has been temporarily suspended [20] or because the PM part of the task is completely finished [21, 22]. Importantly, the PM target appears during the interval in which the participant is not supposed to respond. Bugg et al. observed that some participants still responded to the target (i.e., commission errors) during the phase in which the task was ended [21]. Further, commission errors can be increased with encoding fluency manipulations [21] or highly salient targets [23], thereby showing that those manipulations were likely generating spontaneous or automatic retrieval of the PM intention. In other words, if the retrieval of future intentions is truly automatic or spontaneous, then it would be hard to deactivate the intention, even when the intention is not supposed to be performed. We implemented this paradigm in the current study to further test that retrieval of the PM intention had become automatic.

### The present study

The current study was an exploratory investigation of whether a PM task might transition from resource-demanding to relatively automatic with repeated practice over many weeks. Children who regularly participated in cognitive testing at a daycare were instructed to ask the experimenter to stamp a reward card at the end of each session, with the goal of accruing a certain number of stamps to earn a prize. PM performance was scored using a system that indicated how much prompting was needed to remember the intention after each session. More prompting indicated more resource-demanding retrieval. In general, we expected the amount of prompting needed to remember to ask for the stamp to decrease with practice.

During these trials, we also tested to see whether particular variables, like visual reminders, might influence the success of children’s PM remembering. We predicted that cue visibility would impact remembering based on previous research demonstrating strong effects of reminders and salience [15, 24]. However, we also predicted that the impact of cue visibility would be reduced after participants had developed more experience with the task. This is based on previous work from Kelly et al. [19] which showed that other well-established relationships (e.g., age and PM performance) were attenuated with practice on a PM task (see also Vogel et al. [17]).

Finally, we explored performance when participants were told to no longer ask for the stamp (i.e., suspended or canceled intention). If numerous opportunities to complete the task resulted in the task becoming somewhat automatic, then we would expect to observe that the children in this study may have still asked for the card, even after they had been instructed that the game is over.

## Experiment 1

### Participants

The Georgia State University Institutional Review Board approved this study (Protocol H22281). Written informed consent was obtained from the parents of the children in this research. During testing, children also provided verbal assent. Specifically, each student was asked if they wanted to test on the computers before each session. While verbal assent was not recorded, it was indicated by the child choosing to participate or not choosing to participate. Thus, any session where data was recorded was an indication of assent.

There were 17 participants (60–72 months; 8 females) in the first sample. The average age was 67.4 months (*SD* = 4.29 months). Two of the participants did not finish the study because the school year ended, and these children left the school. The sample was mostly White and from middle to upper-middle-class SES. It is important to note that all of these children were enrolled for long-term cognitive testing with our research group, working with the same research team for many months in a row. Thus, they were all very familiar with the routines of these kinds of tests, although they had never done this particular task and, prior to the current study, had never been asked by the research team to maintain an intended response from one day to the next.

### Materials, design, and procedure

Testing took place in the cafeteria area of the school. All prior testing also occurred in this area, and thus participants were very familiar with this setting. For any given testing session, participants were accompanied back to the testing area to begin working on a task unrelated to the PM task that is germane to the present study. These tasks most often, but not always, involved completing trials of a computer game that assessed some element of the child’s perceptual, attentional, or memory abilities. This is important to note because it means across the study period, children were in a familiar setting but were receiving new and varying primary tasks to complete. Thus, the setting was not completely routinized, and the only consistent aspect was the location. The entire testing sequence is presented in Table 1. Children were tested one at a time. After completing the primary task on the first day of this study, the child was shown a note card along with a small opaque wooden box in which the cards were stored. The note card had a place for the child’s name at the top and then 5 empty circles across the length of the card. The experimenter wrote the child’s name on a card and then explained how the card worked. Children were told the goal was to get five stamps on their card. In addition to the small prize they received for completing each day’s testing on the primary task, they were told that when they had collected all five stamps, they would earn a “super prize.” The participants were informed that the cards would be kept in the box and that at the end of every testing session they should ask the experimenter to give them a stamp on their card “because sometimes we [the experimenters] forget.” Participants experienced two “training sessions” in which the instructions were repeated in this way (Session 1 and Session 2) and they were explicitly reminded of the box and how the stamping system worked. After these two training sessions, test sessions began.

**Table 1:** The sequence of test trials for all three cards of the habitual PM task in Experiment 1.

Trial	Card	Condition	# of Stamps
1	--	Training	1
2	--	Training	1
3	1	Initial Learning	1
4	1	Initial Learning	1
5	1	Initial Learning	1
6	1	Initial Learning	1
7	1	Initial Learning	1
8	2	Proximity to Prize^a^	3
9	2	Proximity to Prize	1
10	2	Proximity to Prize	1
11	3	Non-Visibility^b^	1
12	3	Non-Visibility	1
13	3	Non-Visibility	3
14	--	Suspended Intention	0

*Note*. a. Performance on this trial was compared to Trial 7 to assess the impact of being in close temporal proximity to the reward on PM performance.

b. Performance on this trial was compared to Trial 10 to examine whether making the box invisible would impact PM performance.

### Initial Learning condition

At the start of the third trial, PM performance was intentionally measured (i.e., Initial Learning condition or Card 1; see Table 1). In this condition, when children finished the primary task, the experimenter did not say anything about the box, but recorded whether a child spontaneously requested their card to be stamped. If the child collected their daily prize for completing the primary task and prepared to return to the classroom without mentioning the box, the experimenter initiated a series of scripted reminders related to the box that led to a recorded PM score for that day (see Table 2). Under this rubric, a higher score indicates that the participant needed more specific and direct prompting to remember the intention. With this rubric, children were guaranteed to receive a stamp during every testing session. This kind of scoring rubric has been used previously in PM investigations in children [25].

**Table 2:** Prospective memory scoring rubric. The experimenter always progressed through this series of prompts until the participant correctly requested the box and/or a stamp of the card. Participants’ score was based which prompt preceded their request for a stamp on their card.

Score	Prompt
0	No prompt; participant spontaneously requested a stamp on their card before the experimenter said anything.
1	Participant requested their stamp after the experimenter asked, “is there anything you were supposed to remember to do?”
2	Participant requested their stamp after the experimenter asked, “was there anything you were supposed to remember about this box?”
3	Participant requested their stamp after the experimenter asked, “do you remember what you did last time you picked out a toy?”
4	Participant requested their stamp after the experimenter asked, “do you remember what is inside the box?” This was the highest score a participant could earn on a trial. If they still failed to answer correctly here, we told them what they needed to do, and then gave them a stamp.

For the five trials of the Initial Learning condition, the box was kept on the table during testing, and was fully in view of the child, although it was not placed directly in front of them or directly next to the apparatus they engaged in their primary task (e.g., the laptop computer they might be working on). No matter how well a child remembered the need for stamps, a trial always ended with a stamp being delivered, so that five sessions led to a filled card. After collecting five stamps on Card 1, children were awarded with a super prize (i.e., two choices from a special collection which contained toys and prizes that were not normally available). The experimenter explicitly told the participants that even though Card 1 was complete, a new card was started for next time they worked with the experimenters. We did not stamp the new card during this session, but participants were informed that they should keep asking for the box.

### Card 2: Proximity to prize condition

The first trial of Card 2 was the most critical (i.e., Proximity to Prize condition; see Table 1). Specifically, performance on the last trial of Card 1 was compared with the first trial of Card 2. If children’s improving performance on the PM task was only due to getting closer to the prize, then we would expect performance to drop when Card 2 was started and they were no longer close to earning the prize. However, if patterns of performance remained consistent or improved across subsequent trials, this would suggest that repeated exposure to the task was the primary driver of performance. We refer to this as the “Proximity to Prize” condition and it was our primary interest for analytical purposes regarding this aspect of PM performance.

Trials for Card 2 were conducted the same way as previous sessions with one exception: For the first trial of Card 2, participants were given three stamps instead of the usual one, meaning participants completed Card 2 in only three sessions. Participants were not aware that they were going to receive three stamps until after they retrieved the intention to get a stamp on that first occasion, so that did not influence their performance on the first trial of Card 2. The reason for giving three stamps is because after the “Proximity to Prize” condition, we wanted to quickly move onto Card 3 where we sought to examine the influence of visibility.

### Card 3: Non-visibility condition

After completing Card 2 and receiving their second super prize, participants were once again told that a new card would be started so they should keep asking for stamps. However, for Card 3 (i.e., Non-Visibility condition; see Table 1), the box was no longer visible to participants when they came to the testing area. It was kept below the table and out of the participant’s view. We compared the performance on Trial 1 of Card 3 to the final trial of Card 2.

Additionally, on Trial 3 of Card 3, participants received three stamps. Thus, like Card 2, this card was also completed in three sessions, but children did not know the card would fill to completion until the day on which we did this. Therefore, better performance on this trial could not be attributed to greater motivation.

### Suspended intention condition

After participants earned their super prizes for completing Card 3, they were explicitly told that the card task was complete and there was no need to ask for it any longer (i.e., Suspended Intention condition). During the next testing session, participants came back to complete their normal testing on the primary task. During testing, the box with the cards remained on the table in plain view so as to provide a strong visual cue, although in this case, the cue was invalid. Participants were given a score of 1 if they asked for a stamp or were given a score of 0 if they did not ask for a stamp.

### Results

#### Initial Learning condition

The complete set of participant data for all conditions and trials are online at https://osf.io/458eg/. Table 3 displays the average performance of participants across conditions. Children required 1.47 reminders (*SD* = 1.17) to successfully complete the PM intention on their first test trial (recall that this was after they had completed two training trials). By the fifth trial of Card 1, participants only required an average of 0.47 reminders (*SD* = 0.62). To confirm that this pattern reflected significant improvement, a one-way within-subjects ANOVA was conducted. There was a significant decrease in the number of reminders needed, *F*(4, 13) = 4.58, *p* = .016, partial *η*^*2*^ = .59. An a priori planned comparison revealed a significant difference between Trial 1 and Trial 5 performance, *t*(16) = 3.12, *p* = .007. These results show that children improved in their ability to independently request the stamp and thereby complete the habitual PM intention.

**Table 3:** Participant performance as a function of card and trial on the habitual PM task.

	Initial Learning Condition (Card 1)					Card 2			Card 3		
Measure	1	2	3	4	5	6	7	8	9	10	11
M	1.47	1.12	0.71	0.47	0.47	0.53	0.76	0.47	0.53	0.33	0.4
SD	1.18	0.93	0.77	0.62	0.62	0.62	0.75	0.51	0.83	0.49	0.63

*Note*. M = Mean. SD = Standard deviation.

#### Proximity to prize condition

Next, we assessed performance on the first trial of Card 2 (i.e., Proximity to Prize condition) as compared to the last trial of Card 1. If proximity to the reward was the driving factor for improved performance, the amount of prompting should increase when starting again with a blank card. We found no significant difference between the last trial of Card 1 (*M* = 0.47, *SD* = 0.62) and the first trial of Card 2 (*M* = 0.53, *SD* = 0.62), *t*(16) = -0.25, *p* = .81.

#### Non-visibility condition

For the final comparison, we examined the difference between the last trial of Card 2 (*M* = 0.47, *SD* = 0.52) and the first trial of Card 3 (i.e., box removed from sight; *M* = 0.53, *SD* = 0.83). There was not a significant difference between these two trials, *t*(14) = -0.25, *p* = .81. This suggests that remembering to request the stamps had become sufficiently habitual to allow for good performance even without a visual reminder of the box.

#### Suspended intention condition

After completing Card 3, we carried out a Suspended Intention probe trial in which subjects were told on the previous test session day that they were to no longer ask for the box. Of the 15 children who completed this probe, only three of them requested the box or stamp. A chi-square goodness of fit test revealed this to be a significant difference compared to the proportion of requests for a stamp on the last trial of the Non-Visibility condition in which 10 of those 15 children spontaneously requested the box or stamp without cues from the experimenter, χ^2^(1) = 5.4, *p* = .02.

#### Post-hoc: Minimal experience control group

**Fig 1:**
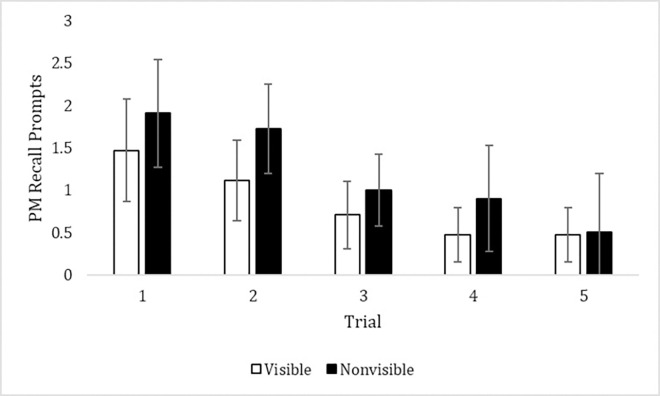
PM performance over the first five trials as a function of visible (*N* = 17) or nonvisible (*N* = 11) Initial Learning condition. Error bars represent 95% confidence intervals.

Given the data, we surmised that visibility did not have an influence because PM retrieval was already routinized. Perhaps placing the box out of view on earlier trials would have created deleterious effects on PM performance. To test this idea, we collected a post hoc set of data with a new sample of 11 children (60–71 months, *M* = 65.2, *SD* = 4.6) who were naïve to the task. After two initial training sessions like those described earlier, these children began the Initial Learning condition but importantly, the box was not visible during these trials for this sample. We used a paired *t*-test to compare the average performance of children from the original sample (for whom the box was visible during the Initial Learning condition) to the new sample of children (for whom the box was not visible during the Initial Learning condition). The results indicated that the nonvisible condition (*M* = 1.21, *SD* = .59) was associated with a higher number of reminders than the visible condition in the initial learning process (*M* = .85, *SD* = .44), *t*(4) = 3.73, *p* = .02, see Fig 1. Thus, when the box was not visible during the first few trials of the task, participants needed more prompting from the experimenter. This result seems to confirm our initial suspicions that the failure of the visual reminder to impact performance was due to the task already becoming somewhat habitual.

### Discussion

Experiment 1 demonstrated that when given numerous occasions to perform the same PM task, preschool students required less prompting as they gained more experience. On the 6^th^ trial (i.e., “Proximity to Prize” condition), which was the beginning of a new card (i.e., Card 2), participants needed very little prompting, suggesting the task had become fairly routine. Similarly, performance was stable during the Non-Visibility condition (i.e., first trial of Card 3), suggesting that the visibility of the box did not influence established performance.

However, this seemed to be because the task was already somewhat automatic. When the initial group of children was compared with a post hoc group for whom the box was nonvisible during their initial sessions, an effect of visibility emerged. However, this visibility comparison was made in a post hoc fashion, with a smaller sample collected at a later time point. We therefore wanted to replicate the effect of visibility more directly and intentionally in Experiment 2.

The results of the suspended or canceled intention sessions provided some initial evidence that habitual PM responding was becoming automatic for children. Specifically, three children reported asking for the box (i.e., commission error) even after they were told they no longer needed to ask for the box. We sought to replicate this finding in the second experiment as well.

## Experiment 2

Experiment 2 operated almost identically to Experiment 1 with one exception. After the two initial training sessions, participants were randomly assigned to the visible or nonvisible condition. After completing 5 trials under that condition and receiving their super prize, each participant was given a second card, and switched to the other condition for 5 sessions. Based on the findings from Experiment 1, we hypothesized that there would be an effect of visibility on the first session of the first card, such that children in the visible condition would require fewer prompts than children in the nonvisible condition. However, by the sixth session (i.e., the first session of the second card), we anticipated that remembering would be fairly routinized, and there would be no difference in the number of prompts required between the two conditions. We once again recorded potential commission errors after the completion of the task.

### Method

#### Participants

Participants were recruited from the same daycare and in the same way as Experiment 1. A new sample of 22 children (ages 49–63 months; 10 females) participated in this second experiment. The average age of the children was 54.6 months (*SD* = 3.5). In order to study a sufficient number of participants, two separate waves of children were recruited. The first wave of students (*N* = 13) were tested starting in Fall 2019 until March 2020. Testing for this cohort had to be halted because the daycare was shut down due to the COVID-19 pandemic. Therefore, 4 students were only able to complete 9 of the 10 sessions and one student was not able to complete sessions 7 through 10. The second wave of students (*N* = 9) were tested starting in October 2022 until March 2023, covering a roughly similar time duration.

#### Materials, design, and procedure

Participants were tested with the same materials and in the same location and context as in Experiment 1. Each participant received two training trials before the scoring protocol was initiated. After the second practice session, participants were randomly assigned to either the visible or nonvisible conditions. Visibility for participants was operationalized the same way as in the first experiment.

Upon completion of Card 1, participants received their super prizes and were explicitly shown a new, blank card with their name on it. They were instructed to keep asking for their stamps when they came for testing so they could earn more prizes. At this point, participants switched conditions.

Upon completion of the 10^th^ session, participants received their super prizes and were told they did not need to remember to ask for stamps anymore because the game was over. In two subsequent sessions, experimenters recorded whether or not participants asked about the box or stamps (i.e., commission errors), even though the task had been ended.

### Results

#### PM performance

**Fig 2:**
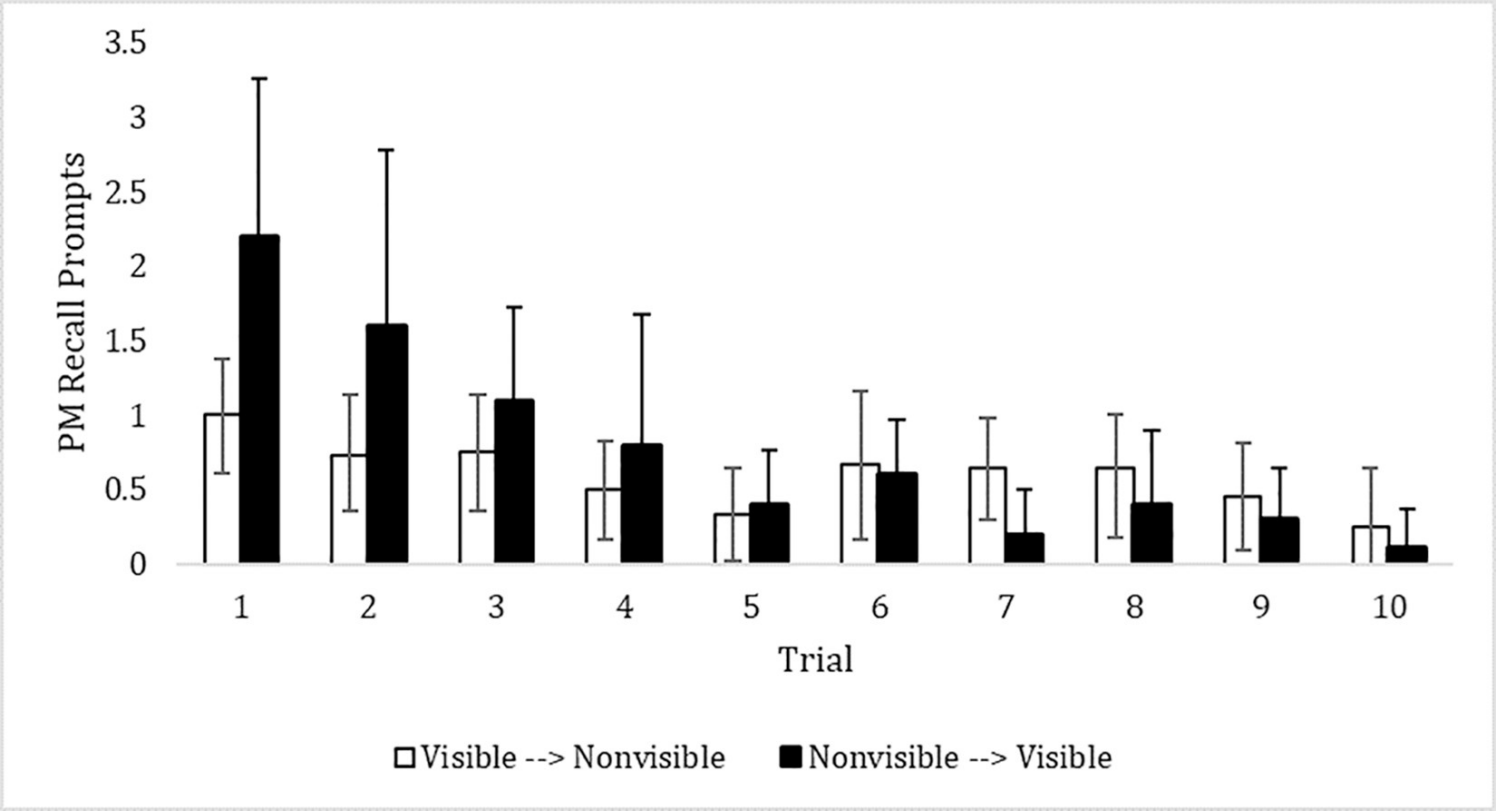
PM performance over 10 trials as a function visibility condition. Error bars represent 95% confidence intervals. Participants switched conditions after the 5th trial.

Fig 2 depicts the average number of reminders needed by participants across sessions as a function of visibility condition. The data replicated what was observed in the first experiment. With experience, preschool children needed fewer reminders in order to recall the intention. To quantify this pattern, we submitted the data to a 2 (Condition: Visible, Nonvisible) x 2 Time (Trial 1, Trial 6), mixed-model ANOVA. There was a main effect of time, such that more prompting was required during Trial 1 as compared to Trial 6, *F*(1, 20) = 11.00, *p* = .003, partial η^2^ = .36. There was also a main effect of condition, *F*(1, 20) = 4.96, *p* = .038, partial η^2^ = .20. This effect stems from individuals in the visible condition (*M* = .83, *SD* = .17) needing fewer prompts than those in the nonvisible condition (*M* = 1.40, *SD* = 1.9). Importantly, these effects were qualified by a significant interaction, *F*(1, 20) = 4.72, *p* = .042, partial η^2^ = .19. The interaction arises from the finding that during the first session, children in the visible condition needed fewer reminders (*M* = 1.00, *SD* = .31) compared to children in the nonvisible condition (*M* = 2.2, *SD* = .34). However, during Trial 6, the number of prompts needed was fewer than Trial 1 and did not differ between the visible (for whom the box was now not visible; *M* = .67, *SD* = .19) and nonvisible (for whom the box was now visible; *M* = .60, *SD* = .21) conditions.

#### Suspended intentions

We recorded the number of times children asked about the box after they had been told the task was finished. These data were not able to be collected during the first wave of data because of school closures due to COVID-19. Thus, data were only available for the nine participants in the second wave of data collection. Seven of the nine children asked about the box during the session following the last session of the task. One of these students also asked about it again, during the second time he went back for testing following the end of the task. The other children did not ask a second time.

### Discussion

The results from this experiment confirmed two primary findings from Experiment 1. First, children were able to more easily initiate prospective remembering on their own as they gained experience with the task. Second, during the first few trials, participants needed more prompts when the box was not visible as compared to when the box was visible. However, this difference disappeared by the time participants reached the sixth session. Finally, the results from the suspended intention condition were similar to Experiment 1. A majority of participants in the second wave committed commission errors. This supports the idea that the retrieval of the intention was so automatic at that point in testing, that they still initiated the task even after they had been told they did not have to ask for the stamps anymore.

## General discussion

The current investigation explored habitual PM in preschool-aged children. Participants had to remember to ask for a stamp at the conclusion of testing sessions in which they had performed an unrelated task. In the first experiment, participants completed this task 13 times (including two training trials) where they were supposed to spontaneously request that they be given a stamp. In the second experiment, participants completed the task 12 times (including two training trials). Our results demonstrated that at first children needed several reminders to retrieve the intention of gaining a stamp. However, with practice, the task became more routinized and children were consistently retrieving the intention without prompts from the experimenters. Although there are few studies that have explored PM through a repeated measures design, these findings are consistent with Kelly et al. [19] who demonstrated that PM performance substantially improved over just three trials in an episodic event-based PM task.

In this habitual PM context, we also examined the influence of cue visibility. Previous research in children indicated that having a visual reminder of the intention improves PM performance [14, 24]. Interestingly, after 8 instances of retrieving the intention in Experiment 1, removing the box from participant’s line of sight had no effect on performance. In other words, the task had become so routinized that variables that normally influence PM performance such as cue visibility did not. This finding prompted the use of a post-hoc control condition and a second experiment to explore if the visible nature of the box would influence habitual PM before it is habitual. The data demonstrated significant differences between the visible and nonvisible conditions on the first test trial. However, these differences were no longer apparent on the 6th trial. For Experiment 2, given that these data were collected during different time periods, we examined whether the same pattern was observed in both waves. The effect of visibility on Trial 1 was apparent during Wave 1 (2.4 reminders vs. 0.88) and Wave 2 (2.0 reminders vs. 1.25). Those same differences were reduced at Trial 6 for Wave 1 (0.8 reminders vs. 0.5) and Wave 2 (0.4 reminders vs. 0.75).

These findings suggest that when a task becomes habitual, visual reminders no longer influence performance to the degree they can in more episodic-like event-based PM tasks. This result aligns with recent research from Vogel and colleagues [17] in adults, which showed that over the course of many trials, PM performance increased along with a reduction in costs associated with responding to the PM target. The results of the current study do not fit neatly with current theories of PM. Tasks in children, like remembering without visual reminders, are thought to require resource-intensive cognitive processes to successfully initiate PM retrieval [11]. Our study demonstrates that there are also mechanisms related to practice, and not specifically related to EFs, that are needed to understand changes in PM development and performance. Unlike Vogel et al. [17], we did not explicitly include measures of cost that might shed light on the underlying mechanisms of this transformation. Several recent PM studies with children have successfully used RT costs to explore the cognitive underpinnings of PM retrieval in event-based PM paradigms [26, 27], and this seems like an important element to include in future investigations of habitual PM in children. It would also be of interest to see if the influence of other manipulations that recruit and require executive function resources, like non-salient or nonfocal targets, would also diminish in a habitual PM context.

Another unique element of the study is the exploration of suspended or canceled intentions in young children. Suspended intentions have been used in the adult literature [21] to explore whether intentions that should no longer be acted upon still activate PM retrieval processes. Our results demonstrated that 3 of the 15 participants in the first experiment asked for the box, even after being told that they no longer needed to do so. In Experiment 2, 7 of 9 participants committed at least one commission error. The presence of commission errors, in general, is further evidence that prospective remembering in later trials was indeed habitual and potentially operating through forms of automatic processing. Consistent with the current data, Cottini and Meier [28] recently examined the performance of slightly older children (6 to 9 years old) with a deactivated intention. While commission errors were very rare, RTs to formerly relevant PM targets were elevated, indicating that there may have been spontaneous retrieval of the intention even after they were told to no longer respond to the PM target.

The suggestion that these commission errors in the current task demonstrate that PM retrieval was automatic is tempered by two possibilities. First, it is possible children may have known the task was completed but asked anyway in hopes of getting another prize. We think this is unlikely because getting a stamp at that point would not have resulted immediately in a prize, and thus there would be no immediate reward for asking. However, we cannot rule out this possibility. Second, these data are from a small sample size and thus should be treated cautiously until further research can be done. Future investigations with children using a suspended intention paradigm could help address these issues.

A potential shortcoming of the present research is that we have a smaller sample size than what is normally reported in research with children. As noted in Smith and Little [29], small *N* designs can in some cases be as powerful and informative as large *N* designs. A post hoc power analysis conducted using G-Power determined that given our effect size and sample size, our power to detect differences in prospective remembering was greater than .99. We believe having a small sample size, but repeatedly testing these individuals over several months can be an important complement to the more traditional designs in which a larger number of participants are sampled at a single time point. This repeated measures approach represents an important perspective to fully understanding the development of PM and other cognitive abilities across the lifespan. Of course, extending this task to children of other ages, from different cultures, different socio-economic backgrounds, and other factors (e.g., outside of the school setting) that contribute to individual differences in performance will be important.

In summary, we found that young children can learn to perform habitual PM tasks relatively quickly. At first, participants needed some reminding to perform the intention. Additionally, at these early stages, participants were negatively influenced by not being able to see a visual reminder of the intention in their line of sight. However, with consistent practice, participants demonstrated an ability to remember to perform these intentions with little prompting from the experimenter. The ability to perform the task remained consistent even as the proximity to a reward or visibility of the target changed. This study represents an initial investigation into habitual PM, however, more research is needed to further explore the dynamics of how this kind of task develops.
